# Food Environment Assessment in Primary Schools Before the Implementation of Mexico’s 2025 School Food Guidelines: A Mixed Method Analysis

**DOI:** 10.3390/children13010088

**Published:** 2026-01-06

**Authors:** María Fernanda Rodríguez-Hernández, Ana Cecilia Fernández-Gaxiola, Larissa Betanzos-Robledo, Paola Guadalupe Ligonio-Gamas, Daniel López-Camarillo, Daniela María Tanchez-Sandoval, Sandra Jocelyn Mejía-Becerril, Verónica Noemí Álvarez-Rojas, Alejandra Cantoral, Esther Nissan-Schoenfeld

**Affiliations:** 1 School of Public Health of Mexico, National Institute of Public Health, Cuernavaca 62100, Mexico; maria.rodriguez@insp.edu.mx; 2 Research Center for Nutrition and Health, National Institute of Public Health, Cuernavaca 62100, Mexico; anafdezg@hotmail.com; 3 Doctoral Program in Epidemiology, National Autonomous University of Mexico, Mexico City 04510, Mexico; larissa.betanzos@comunidad.unam.mx; 4 Master Program in Applied Nutrition, Iberoamericana University, Mexico City 01219, Mexico; a2042415@correo.uia.mx (P.G.L.-G.); daniel.lopez07@correo.uia.mx (D.L.-C.); a2279855@correo.uia.mx (D.M.T.-S.); a2280730@correo.uia.mx (S.J.M.-B.); veronia.alvarez03@correo.uia.mx (V.N.Á.-R.); 5 Health Department, Iberoamericana University, Mexico City 01219, Mexico; alejandra.cantoral@ibero.mx; 6 Postdoctoral Program in Artificial Intelligence for Public Administration, National Institute of Public Administration, Mexico City 04510, Mexico

**Keywords:** school, ultra-processed food, mixed methods, school food guidelines, school food environment

## Abstract

**Highlights:**

**What are the main findings?**

- There is high availability and consumption of ultra-processed foods (UPFs) and sugar-sweetened beverages (SSBs), and limited access to safe drinking water in Mexican schools.
- Principals and teachers are aware of the School Food Guidelines (SFGs) but recognize barriers to their implementation, such as resistance from parents and students to adopting healthier eating habits, the economic dependence of school cooperatives on the sale of UPFs, and insufficient infrastructure in public schools to support the preparation and sale of nutritionally adequate foods.

**What are the implications of the main findings?**

- Reinforce comprehensive and sustainable strategies for the proper implementation of SFGs that include schools, families, and the community, and establish a system to monitor the implementation of SFGs.
- Invest in school infrastructure that allows for the provision of healthy food and safe drinking water.

**Abstract:**

Background: Childhood obesity and being overweight represent a global public health challenge; the consumption of sugar-sweetened beverages (SSBs) and ultra-processed foods (UPFs) contributes to this problem. In Mexico, public health policies have been implemented to improve school food environments. Objective: To assess the school food environment before the implementation of the guidelines for the preparation, distribution, and sale of food and beverages (SFGs) in three primary schools in Mexico City. Methods: A cross-sectional mixed method study was conducted, including structured non-participatory observation of selling points outside and inside of schools, and availability of drinking fountains and lunchboxes contents. A food waste audit assessment was performed to identify the most frequently consumed products, main ingredients, front-of-package labels, and colorants. Additionally, 23 semi-structured interviews were conducted with school authorities, teachers, and food vendors. Results: SSBs and UPFs selling points were observed outside and inside in public schools, while in private schools, only inside, with use of delivery food apps. Public schools lacked functional drinking fountains. A total of 345 food waste items were collected across the schools, of which 46.3% were SSBs and 53.7% were UPFs. The main ingredient was sugar (15.6%), the principal front-of-package was excess sugar (37.5%), and the most frequently used colorants were red 40 (25.1%). Interview participants reported awareness of the SFGs; however, they identified barriers such as resistance from parents and students and the economic dependence of school cooperatives on UPFs sales. Conclusions: These findings highlight structural and economic challenges for the effective implementation of public policies promoting healthier school food environments.

## 1. Introduction

Childhood overweight and obesity (OW/OB) pose a significant global public health concern. According to the World Health Organization (WHO), more than 390 million children and adolescents aged 5–19 years were overweight in 2022, and of these, 160 million lived with obesity; a prevalence that has tripled since 1990 [1]. In Mexico, data from the 2023 National Health and Nutrition Survey (ENSANUT) reported that 18.5% of school-aged children (5–11 years) presented overweight, while 15.7% obesity [2]. The consumption of sugar-sweetened beverages (SSBs) and ultra-processed foods (UPFs) high in added sugars, saturated fats, sodium, and additives has been strongly linked to the development of OW/OB and other chronic diseases [3,4]. Particularly, the intake of low-nutrient, energy-dense foods is adversely associated with body mass index, weight, and intake of energy among children and adolescents [5,6].

The Food and Agriculture Organization of the United Nations (FAO) establishes that the school food environment refers to all spaces, infrastructure, and circumstances within school facilities where food can be found, obtained, purchased, or consumed, as well as its nutritional content [7]. In Mexico, the first recorded regulation of the school food environment and encouragement of physical activity to control OW/OB was the establishment of the National Agreement for Nutritional Health (ANSA) in 2010 [8,9]. However, due to the non-obligatory nature of this regulation, which is limited to being an agreement between institutions, and without clear criteria on the nutritional quality of food, it did not have the expected impact, as reported by the Mexican non-profit civil association. “The Power of the Consumer” indicates that the goals set out in this document for 2012 were not met [10]. In 2014, a tax on sugary beverages was implemented as a strategy to reduce SSBs consumption and manage the development of chronic noncommunicable diseases [9,11]. In addition, the ANSA agreements were reactivated and published in the official gazette of the federation this year, establishing them within Mexican normativity, but without clear sanctions or monitoring mechanisms [12]. Finally, in October 2020, the front-of-package warning labels were instituted [13]. However, despite these measures, records indicate that 98% of schools continued to sell UPFs and SSBs during the 2023–2024 school year [14]. Since the previous government measures to establish healthy school food environments proved unsuccessful (ANSA 2010 and 2014), the actual Mexican government published new guidelines on 30 September 2024, but it was not until 29 March 2025 that these came into effect, with the main objective of establishing the provisions governing the preparation, distribution, and sale of prepared, processed, and bulk foods and beverages, in accordance with nutritional criteria, in all schools within the National Education System [15], which, for this study, will be abbreviated as School Food Guidelines (SFGs). The difference with previous measures is that these SFGs are mandatory as they have been published in the General Education Law, establishing penalties for schools that fail to comply with these measures, as well as prohibiting the sales of foods with warning labels, being clearer about the type of foods prohibited, including the concept of sustainable food, and active monitoring with an evaluation every 5 years [15].

It has been recognized that school nutritional education and other activities to promote healthy eating and wellness can change the school food environment [16] as children spend a significant amount of their time in the school setting, and, on average, it has been reported that 35% of their daily food intake occurs at schools [17,18].

Assessing the food environment in schools before implementing a program allows for the identification of current conditions that influence students’ eating behaviors, including the availability, accessibility, and promotion of foods within and around the school. This assessment provides a baseline to understand the physical, economic, sociocultural, and regulatory factors that shape food choices, as well as the barriers and opportunities to improve the availability of healthy options. Such information supports the design and implementation of more context-specific, effective, and sustainable interventions, while also generating evidence to inform decisions and policies aimed at promoting healthy school environments [19,20]. Therefore, this study focuses on evaluating the food school environment using mixed methods before the implementation of the SFGs in three primary schools in Mexico City. Particularly, we aimed to assess food offered inside and outside of schools, access to drinking water, the type of lunchboxes consumed, and waste food audit assessment, as well as explore the perceptions among school principals, teachers, and food sellers about the SFGs.

## 2. Materials and Methods

### 2.1. Study Design

A cross-sectional, mixed method study was conducted in three primary schools in Mexico City (children aged 6 to 11–12 years), combining quantitative and qualitative methods to obtain a more complete understanding of the school status before the implementation of the SFGs, during the period of March–April 2025. Two were public primary schools (identified as S1, S3) located within the Álvaro Obregón municipality in the town of Santa Fe with a matutine school schedule, and one was a private primary school (S2) in the Cuajimalpa municipality with an extended school day program. The schools were selected through convenience sampling based on pre-existing collaboration with the community center “Centro IBERO Meneses” from Universidad Iberoamericana, derived from a principal study conducted in 2023, where the methodology for this study was piloted. Schools had not implemented SFGs at the time of data collection, as we visited them before its full implementation. The principals mentioned that the changes stipulated in the SFGs had not been implemented on the day of school visits.

### 2.2. Data Collection Procedures

Food environment assessment combined qualitative and quantitative approaches for understanding the availability, accessibility, promotion, and consumption of food in a context [21]. Data collection included structured non-participatory observation, food waste audit assessment, and semi-structured interviews with principals, teachers, and food vendors. All procedures were carried out before, during, and after lunchtime by trained personnel and are described below.

#### 2.2.1. Structured Non-Participatory Observation

We conducted structured non-participatory observations with the aim of systematically and objectively recording information. The main constructs observed were as follows: (1) The food offered inside and outside of schools (we included aspects such as food vendors inside and outside within the immediate school perimeter of two blocks around). The type of products sold was recorded, and food advertising or branding within school facilities was documented. (2) Access to drinking water; we observed whether students had access to safe drinking water through school drinking fountains, water jugs, or other sources, including the functionality and hygiene conditions of drinking fountains. (3) Lunchbox visual analysis; during lunchtime, we analyzed a subsample from lunchboxes of 10% of children who attended the school visit day, and recorded the brands, types of products, and packaging materials. These observations were complemented by a photographic record that was later analyzed by the research team. In the case of school S1, we did not obtain permission from the school authorities to take photographs of the students’ lunchboxes, but we did capture the above-mentioned information in writing records only.

#### 2.2.2. Food Waste Audit Assessment

The food waste audit assessment was used in this study because it provides a non-reactive measure of consumer behavior through the analysis of material waste. This method yields objective information that complements the qualitative findings by reflecting actual dietary practices, as individuals are unaware that they are being evaluated, and therefore do not alter their behavior [22]. We conducted a food waste audit following the Garbage Project methodology, which was developed at the University of Arizona for Dr. Bill Rathje to objectively evaluate feeding patterns and behavior toward waste using archeological methods, offering the advantages of being a non-reactive measure of behavior, low cost, and not requiring the participation of study subjects. The methodology consists of classifying, coding, and recording the objects found in the trash, where the classification of the trash depends on the study objective, which may include the weight of the materials, without registering personal information [23,24]. Upon arrival at each school, a record of the number of students attending on the day of the visit was provided by the principal. At the beginning of lunchtime, bags were placed next to the trash cans to collect the waste discarded by students. At the end of recess, staff equipped with plastic gloves organized all the waste generated by product type. For example, all apple juices of the same brand were grouped and counted. Photos of the labels were taken to later conduct a detailed review of the nutritional information and product labeling. Once the analysis was completed, the waste was disposed of in the trash cans.

#### 2.2.3. Semi-Structured Interviews

Semi-structured interviews were conducted by trained researchers in qualitative methods with school principals, teachers, and food vendors to explore awareness of the SFGs and their perceptions regarding its implementation and of the school food environment. A purposive sample of principals, teachers, and food vendors present on the day of the school visit was invited to participate; participation was voluntary and required written informed consent. Recruitment continued until thematic saturation was reached [25]. A short guide based on the Health Belief Model (HBM) [26] and the Theory of Planned Behavior (TPB) [27] served as the conceptual basis for identifying perceived barriers and facilitators. The guide was piloted with two volunteers before the start of the study and revised based on their feedback. The final version of the guide included eight open-ended questions. Interviews were conducted inside the schools by trained researchers and were audio-recorded using a digital recorder. The average of interviews was approximately 12 min.

### 2.3. Data Management and Analysis

The information obtained through the structured non-participatory observation guide was grouped and analyzed through contextual interpretation [28]. Quantitative data from the food waste audit assessment were entered into structured spreadsheets and analyzed using descriptive statistics through frequencies and proportions, and Fisher’s exact test was used to determine statistically significant differences between schools. A value that was ≤0.05 was considered a statistically significant value. For each product, labeling information, the five main ingredients, the three main colorants, the front-of-package warning labels, and the type of product materials were recorded. Products were then classified into four main categories: (1) beverages, (2) pastries, cookies, and sweet cereals, (3) snacks and chips, and (4) candies, chocolates, and jellies. We determined the proportion of products per student by dividing the number of food waste items in each school by the number of children attending on the day of the visit. All quantitative results were presented graphically, showing the frequencies and proportions, and all analyses were performed with the program StataNow/BE 18.5 (StataCorp LLC, College Station, TX, USA).

Semi-structured interviews were transcribed verbatim by trained researchers in qualitative methods in a main document and coded thematically to identify key patterns in participants’ perceptions, including barriers, facilitators, and awareness of the SFGs. Systematization and interpretation of the information were guided by the content analysis technique [29]. We developed a priori central themes and aligned them with our research questions before data collection. Information was analyzed independently by the interviewers. Researchers reached conclusions using the peer-review technique, which involved meeting on average five times to discuss results to reach a consensus [30]. Interpretations reached by researchers were presented in narrative form in the results section, accompanied by example quotes from the qualitative data.

### 2.4. Ethical Considerations

The study received approval from the Academic Council (session No. 145) and the Ethics Committee of Universidad Iberoamericana (CEI: 1/23). Written informed consent was obtained from school authorities, teachers, and vendors interviewed. No verbal information was collected from school children. Data were anonymized and stored securely following ethical research standards; schools were identified using codes, and interview records contained no personal information.

## 3. Results

### 3.1. Food Offered Inside and Outside of Schools

Through Structured Non-Participatory Observation, we identify the availability of products inside and outside the schools, S1 was close to a principal avenue with commercial food stores such as bakeries, ice-cream shops, and small grocery stores, as well as a public market. The most frequently observed products included chips and SSBs such as juices, soft drinks, dairy products with added sugar, water bottles, candies, and chocolates. Inside the school, tortilla-based foods (typical Mexican food), ice popsicles, and water sold by the school were available. S2 had no offer of foods outside; however, this school had the widest variety of products available in its cafeteria such as fried food, pastries, typical Mexican food, coffee, chips, cereal bars, and the option to order food through delivery apps. Outside S3, there was one grocery store near the school. Inside S3, there was a small table of Mexican stews made from tortillas, nopales, rice, and beans, as well as fresh fruit. In S1, we observed that food was served in unicell and plastic bags; in S2, the vendors used disposable paper plates and cups; in S3, the stews were offered on reusable plates, which students gave to the food vendors at the end of recess. In general, we observed that the range of fruits and vegetables was scarce. A detailed of example of photographic record is available in Supplementary Figure S1.

### 3.2. Access to Drinking Water

About drinking water access in schools, we observed that all schools had drinking fountains. However, only in S2 were the drinking fountains in good condition, clean, and used by the students. On the other hand, in S1 and S3, despite having drinking fountains, these were not working, so students only had access to water if they brought it from home or bought it. Supplementary Figure S2 shows an example of a photographic record.

### 3.3. Lunchbox Visual Analysis

According to the visual inspection of the contents of the lunchboxes of the students at the time of the visit per school (for S1, we did not obtain permission; for S2, 48 items; for S3, 18 items), we observed that, in general, the most frequent food groups were beverages, such as water bottles and thermos, as well as juices and dairy products with added sugar, chopped fruits, plastic bags with chips, and sandwiches. In terms of schools, we found differences between public and private schools in terms of the variety of products. In private schools, lunchboxes presented a variety of products of brands not available in the Mexican market (mainly Kosher products), and it was the school where lunchboxes had the most variety of UPFs. Additionally, we observed that most of the children of S1 carried a lunch consisting of juice, yogurt with added sugar, a sandwich or hamburger, and fries sold outside of school; however, no photographic record of these packages was obtained. Supplementary Figure S3 is an example of a photographic record.

### 3.4. Actual Dietary Practices

Based on the food waste audit assessment, Table 1 presents a general overview of the three schools evaluated. The average attendance of students on the day of evaluation was 89.5%. In total, 345 items were collected after lunchtime in the three schools, which represents a mean of 0.33 products per student. In Supplementary Figure S4, it shows an example of a photographic record.

**Figure 1:**
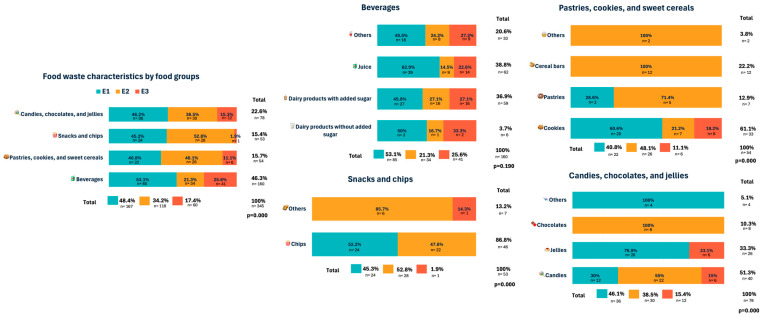
Food waste characteristics by food group and the most consumed food waste item type by food group.

In Figure 1, according to the food group categories, we observed that beverages (46.3%) was the most frequent food waste category, followed by candies, chocolates, and jellies (22.6%), which was in equal proportion to pastries, cookies, and sweet cereals, and snacks and chips (15%) (Figure 1). Regarding the category of beverages, juices (38.8%) and dairy products with added sugar (36.9%) were the most frequently found in the garbage deposits. In the category of pastries, cookies, and sweet cereals, cookies were the most representative of the group (61.1%). For snacks and chips, chips (86.8%) were the predominant element in the group. And for candies, chocolates, and jellies category, candies (51.3%) were the most frequent. The differences according to food group categories between schools were statistically significant (*p* = 0.000). However, inside the group for beverages, the food categories were not statistically different between the groups (*p* = 0.190).

#### Information from the Food Labels

Regarding information from the food labels, the analysis showed some statistically significant differences between the schools studied in terms of the composition of the UPFs. About ingredients, we observed that sugar (intrinsic to food and added sugars) was the most frequent ingredient identified (15.6%), followed by water (8.2%), fruits (8.1%), and milk (7.1%) (*p* < 0.05). About colorants, the most frequent type of colorant was red 40 (25.1%) (*p* < 0.05); in contrast, yellow 5 (20.7%) showed no statistically significant differences between schools (*p* = 0.1). Likewise, significant differences were identified in the type of front warning label, with the most frequent labels for excess of sugar (37.5%) and excess of energy (32.7%) (*p* < 0.05). As with single-use materials, polyethylene/polypropylene was the most frequent (40.6%), followed by tetra packs (25.5%) and plastic containers (20.6%) (*p* < 0.05). According to distribution, school S1 was the one that showed the most frequent consumption of ingredients, colorants, front warning labels, and plastic containers, followed by S2, and S3 showed the lowest frequency. (Supplementary Material Figures S5–S8).

### 3.5. Awareness and Perceptions About the Implementation of SFGs and the School Food Environment

A total of 23 participants were interviewed (Table 2). All participants referred to were aware of the SFGs and one teacher referred to knew the SFGs since they were first published in 2010 [8]. Principals and teachers referred to had other projects in the curricula for promoting health in all grades, so children knew to be careful with what they eat. Some of these projects involve children preparing their own lunchboxes at home or food preparations at school and recognizing the benefits of eating healthily and the consequences of not doing so.

The principals had different perceptions on the SFGs. One principal mentioned that the SFGs are not new, and they have implemented them for a long time but they have never worked. The others mentioned that they were useful, and they spoke to parents about them.

*“In meetings [with parents], we told them about the new law that was going to be implemented, and they signed a document stating their agreement to change their diet accordingly. So, they will support us from home, avoiding sending that type of thing.” (Principal, school 2)*

In one school, teachers referred to had great support and information. On the contrary, teachers in the other two schools referred to had a great need for more information and for dialog with parents. For teachers, there was a general consent in promoting the message of avoiding processed foods and engaging in physical activity, but they also mentioned that it is rarely carried out. Some teachers also mentioned that parents feed their children very poorly and that it was not up to them as teachers. Others referred to said that parents must be involved as they are the role model for their children and yet they reject many changes frequently.

*“The truth is, (the principal) has been a great support. She is filled us with information about this situation, talked with the children, made posters so they avoid junk food and raised awareness among them.” (Teacher 1, school 3)*

When we asked teachers what barriers they foresee in implementing the SFGs, they stated that parents and children themselves are the main barriers. Parents, because they are the ones responsible for their children’s food habits; they buy and prepare their food. Children, because they like UPFs and they are used to it, and parents protect them even when this means letting them eat what they want. No other direct barriers were mentioned by the principals and teachers.

*“The point is to raise awareness, especially among fathers, because we have done our part here, but there are fathers who flat-out said, ‘No.’” (Teacher 2, school 1)*

In the case of food vendors, many perceptions were documented. Vendors referred to said that selling food is part of the school’s ‘cooperative income’ and is used to fix and buy things needed inside schools. This income also allows them to pay for the transportation needed, particularly as these schools are in a disadvantaged and marginal geographical area, and they need to travel some miles for some goods and food. In school 3, the principal reached an agreement that the income provided to the school from selling processed foods will be reduced.

*“The cooperative’s income allows us, for example, who are here in the hole, to go to the area, where we have to take a bus and all that.” (Food vendor 1, school 3)*

*“Selling fruits and vegetables, whole grains, and legumes is not going to be enough. No. No. That money is not going to be enough, and the kids are not going to want to buy that.” (Teacher 4, school 3)*

Although these schools do not have a kitchen or special place for food preparation, these schools do provide a cold breakfast for children a couple of days per week, usually fruit, milk, and cookies, as part of a social assistance program. This benefit is not universal, so it is important that schools have food available for sale. Some children have told their teachers they forgot to have breakfast and/or are hungry.

*“Not every day, but they do get to drink milk, cookies, or sometimes biscuits, fruit, because there are those who do not like milk.” (Teacher 4, school 3)*

Vendors sell UPFs, but also, they sell foods prepared at their homes, due to the lack of kitchens in schools. In one school, a vendor mentioned she was trying to include more vegetables and healthy foods. She mentioned that some children are learning to eat healthier and prefer to eat at school.

*“The children are learning something they did not have before, that habit of eating healthy. They are already learning it, and the principal is supervising them.”*

*“I have seen that everything is the type of food they can eat, and the truth is, very few things are junk food; in fact, they bring junk food from home.”*

A couple of teachers also mentioned a red light from food vendors outside the school. Even when schools implement the SFGs and stop selling UPFs, children will still be able to buy them outside the school every day.

## 4. Discussion

This study provides an integrated assessment of the food environment in three primary schools in Mexico City before the mandatory implementation of the SFGs. In this structured non-participatory observation, we observed that public schools have the widest variety of stores with UPFs outside the schools compared with private schools. Inside the schools, for the public primary ones, typical Mexican dishes were offered; meanwhile, the private primary school offered more UPFs inside, with the option of a delivery app system available (especially for those children who remain after hours). All schools have drinking fountains, but only the private primary school (S2) had clean and functional ones. About children’s lunchboxes, these mainly included water, SSBs, as well as sandwiches, fruit, and chips, with the private school having the most variety of UPFs lunchboxes. Regarding food waste audit assessment, we found 345 items, and some characteristics evaluated were statistically different between schools, with S1 being the school with the most items per student (0.46) intake of UPFs, with the most variety of ingredients, colorants, frontal warning labels, and plastics. Beverages (46.3%) and candies (22.6%) were the most frequent categories, with juices (38.8%) and dairy products (36.9%) being the most reported. According to the ingredients, sugar was the main ingredient (15.6%). About colorants, red 40 (25.1%) was the most frequent. In addition, excess sugar (37.5%) and excess energy (32.7%) were the most frequent front labels in the UPFs, and the most frequently recorded materials were polyethylene (40.6%) and Tetra packs (25.5%). Furthermore, based on the interviews, all participants demonstrated a general knowledge of SFGs, although with divided perceptions about their effectiveness. The main obstacles identified according to principals and teachers’ perceptions for proper implementation were of resistance from parents and children to the guidelines, the lack of school kitchens in public primary schools, and the economic dependence of school cooperatives on the sale of UPFs (on the day of the visit to public schools, UPFs were not offered) with interviewees mentioning that family participation and sustainable economic alternatives are needed to reduce exposure to UPFs in schools.

Consistent with our results about UPFs sales, previous evidence about the school food environment and the sale of UPFs outside of schools, a study carried out in two cities in Mexico found a total of 246 street vendors, 103 stores, and 177 food establishments, mainly around public schools. Street vendors (85%) offered unhealthy foods, such as fried foods, sweets, and SSBs [31]. Similarly, findings have been reported in Brazil, such as an article conducted in public schools (*n* = 36) and private schools (*n* = 34), that showed that there were 285 establishments around schools, with 80% being formal establishments and 20% being informal establishments, all of which were within 500 m of both public schools and private schools. The amount of UPFs sold within the perimeter of the schools (median 18 [12; 21]) was significantly higher than that of fresh/minimally processed foods (2 [1; 4]) and processed foods (0 [0; 1]) (*p* = 0.0001). However, no statistically significant differences were found between public and private schools in any food category [32]. 

This is important because according to the Mexican National Health and Nutrition Survey (ENSANUT) 67.1% of school children consumed more than the international recommendation of 10% of total energy from added sugars, representing around 21.9% of the total energy of this population, and 86.1% consumed SSBs and 55.9% consumed snacks and desserts, with low consumption of vegetables (28.4%) [2]. Similarly, an article stated that among Mexican children, energy from UPFs increased from 27.3% to 30.1% [33], and another Mexican author reported that the consumption of SSBs and UPFs was the principal source of energy among school children in Yucatán, Mexico [34]. Therefore, this reflects the challenge of implementing SFGs due to the wide variety of UPFs and SSBs inside and outside schools, thus establishing a healthy school food environment.

Contrary to our results, other authors have reported a higher obesogenic environment in private schools and attribute these differences to public nutrition policies that public schools are aligned with in countries such as Brazil and the United States [35,36]. For example, a study with a representative sample of 1247 Brazilian public and private schools shows that private schools had an obesogenic environment with a higher prevalence of UPFs and SSBs sales both inside and outside schools, as well as more UPFs advertising and vending machines compared with public schools [35]. And in the United States, a study with national representation (1830 public and private schools), showed that public schools had better conditions in terms of nutrition than private schools, where unhealthy products were limited by 41.6% compared to 27.4% in private schools, with no contracts to promote SSBs and an improvement in the quality of lunches (66.1%) with more fruit included. Overall, the school food environment score was higher in public schools with 53.5 compared to 42.2 in private schools, exceeding by more than 10 points, according to the authors, due to US Department of Agriculture (USDA) standards that lead to control of the food offered to students [36]. 

About water fountains in schools, a cross-sectional study in schools of Northwest Mexico found that 93.3% of 119 schools had drinking fountains, but only 16.2% were clean and functional [37]. Similarly, another article, found in six primary schools in Mexico City that water from the drinking fountains was not fit for human consumption due to lack of cleaning and maintenance; and children did not drink water from the drinking fountains (>80%); the bottled water was also scarce within schools but common at street stalls (19.7%) [38]. This fact leads to selling bottled water for the public schools; as shown in our studio, they showed that they did not have adequate installations as an option for drinking water, so it must come from home, including thermos or bottles with water in the lunchbox. It also presents the challenge of improving the school food environment with free potable drinking water, with the implementation of the new SFGs.

Additionally, we detected different packaging materials in the form of polyethylene terephthalate (PET) and polypropylene (PP), which are susceptible to mechanical abrasion, thermal exposure, and chemical leaching, thereby releasing microplastics into food [39]. The chemicals in the packaging materials such as BPA and phthalates are considered obesogenic chemical disruptors that can affect weight gain through different metabolic pathways [40]. In addition, microplastic migration is also influenced by food composition, with fatty and acidic foods accelerating polymer degradation due to their chemical interactions with packaging materials [41]. In the case of colorants, we noticed that the main ones were red 40 and yellow 5. Recently, a lot of attention was focused on artificial colorants in processed foods as they are linked to a series of health problems in children, including allergies, gastrointestinal, and respiratory problems, in addition to behavioral changes [42].

Although in Mexico, actions have already been taken to reduce the sale of UPFs and their promotion within the National Education System [8,12], as evidenced in our results, adequate implementation has not been possible. This could be due to the different conditions that the two types of schools have in the country, where public schools have less willingness on the part of parents to communicate about their children’s diets, less capacity to offer healthy foods due to dependence on cooperatives managed by personnel outside the school (since these cooperatives are a source of income for improving school facilities, parents support their food service program or school activities in public schools, as previous reports have mentioned [43,44]), inconsistent activities related to adopting healthy habits, and greater availability of stores that sell UPFs before the school starts and after it ends compared with private schools, showing the disadvantage of public schools in terms of access kitchens schools and lack of functional drinking fountains. On the other hand, although private schools showed greater institutional support for establishing channels of communication with parents, they face challenges in terms of controlling the quality standards for the menu offered to students, the availability of clean and safe drinking water, and needing greater internal organization to align with SFGs. The analysis of lunchboxes revealed that exposure to UPFs begins in these children’s homes due to the wide variety of these products in children’s lunchboxes, demonstrating parents’ unwillingness to accept the changes promoted by the school and SFGs. Consistently, previous studies identify the following main barriers for the implementation of programs and interventions: lack of knowledge and interest from parents and students; poor supervision by authorities; cultural patterns that are not conducive to healthy eating [37,38]. An article showed that parents are responsible for providing UPFs food to their children, and even if parents are aware of tools for healthier shopping such as front labeling, this alone does not encourage them to buy healthier products; rather, the cost and convenience have a greater influence on food choice [45]. Therefore, the disadvantage in infrastructure, the lack of school kitchens, lack of clean and safe drinking fountains, the economy, and the disposition that public schools have compared to private schools play important roles in the adequate implementation of SFGs.

The limitations and strengths of our study are described below. First, as a strength, the study utilizes a novel method of food auditing that provides non-reactive information on consumer behavior, which means that student behavior regarding consumption of UPFs and SSBs did not change because they were unaware that they were being evaluated, in addition to the evaluation and pictures of the lunchboxes that explain the patterns better than performing a recall. However, we recognize that the cross-sectional design of the study does not allow us to evaluate the typical behavior of the population studied and it did not obtain the contents of trash cans inside classrooms, which could underestimate our results, in addition to the small sample size, because we only visited three schools by convenience, and all schools were in Mexico City; this could be an issue as it does not represent the situation in all schools in the city or municipalities. However, it was shown that in some schools located in the country’s capital, despite previous SFGs, these continue to be unfulfilled. But as a strength, we selected not only public but also a private school that allowed us to make a comparison between the two settings. On the other hand, although we have obtained some information on parental behavior from school principals and teachers, we recognize it as a limitation as we did not interview parents. We propose that future research could incorporate parents’ perceptions because a previous review about school food interventions documented that involving parents in the intervention increases effectiveness [46,47]. However, the analysis of the lunchboxes provides insight into parents’ education and their level of involvement in changing eating habits of children’s behavior. As a strength of this study, the use of semi-structured interviews enriched the quantitative analysis by exposing the barriers and opportunities for improving the efficiency of the implementation of future interventions.

Since 2010, Mexican stakeholders have been trying to change the food environment of the schools, because the evidence shows that policies designed to influence the school food environment have been successful in changing dietary behaviors and are key to improving it [48,49]. One example of this is increasing potable water availability, providing more fruits and vegetables, and including more local products [8]. The fact is that even though some actions have been released due to the soda tax or the front label warning signs [11,13], schools still had more than 50% of ultra-processed foods for lunchtime [14]. Additionally, contrary to other countries, in Mexico, the cafeteria concept within the schools with a complete food service of hot meals is almost nonexistent. This results in only having two options for lunch in the school context: one is buying food in something like a small store, and the other is that parents send a lunchbox with food to the children. This last point makes our analysis unique, as we also pictured and observed which types of foods came from the houses.

In general, the study has significant strategic value, as it provides baseline evidence and documents the situation before the implementation of the SFGs. This is key to assessing their subsequent impact and identifying gaps and areas where regulation needs to be strengthened. In addition, it helps us to highlight the magnitude of the problem by showing that the presence of UPFs in schools was widespread and that students had easy access to products of low nutritional value, which supports the need for stricter regulations. The results can guide improvements in public policy design and highlight necessary adjustments to the SFGs, as well as enable more effective supervision in schools. The study contributes to the promotion of healthy food environments by providing relevant information for the design of healthy school programs, developing nutrition education campaigns focused on the real needs of schools, and providing elements for the development of school staff training programs. In turn, the study can reinforce existing public policies in Mexico, such as food labeling and taxes on sugary drinks and unhealthy foods. By documenting the availability of UPFs, pressure is exerted to strengthen oversight and accountability, as well as to improve the regulation of school cooperatives, making decisions based on scientific evidence.

For future research, we suggest conducting a longitudinal study to assess changes before and after implementing the SFGs. This will help observe the level of compliance over time, verify if the reduction in UPFs in schools is genuine, track changes in students’ consumption patterns at school, and assess whether there are differences between the consumption of children and preteens. Furthermore, monitor the degree of adoption and adaptation, where applicable, of the SFGs. Another suggestion for the future is to expand the sample and the diversity of schools (across states and regions of the country, and urban and rural schools). This would allow for a comparison of how product availability varies according to context. In the future, it is suggested to evaluate factors that favor or hinder compliance with the guidelines, above all, including the perception and training of administrators, teachers, and cooperative staff, and especially parents. Including parents in the research will be important, since, as the article mentions, parents are responsible for selecting the snacks they send their children to school with. Moreover, conduct a randomized controlled trial with a small sample size, where each school adds one of the four points specified above to compare implementation conditions and feasibility.

Experiences from different countries have shown that the setting of effective, healthy, and feasible school food guidelines requires continuous improvement (for example, Brazil and Ghana) [50,51]. This means there is a need to gradually aim to optimize according to needs and set a model with periodic revisions to integrate new evidence and findings [52], for example the ANSA form 2010 was institutionalized but not enforced beyond the government mandate [8]. SFGs need to be inclusive, coherent with the rest of the school food environment and potentially with the home, being iterative and sustainable. To achieve all these, and to be sustainable, there is a need to pilot, improve, and implement. Being transparency and free from conflicts of interest is fundamental [53]. Although SFGs are context-specific, whether they are limited to selling foods, or providing snacks, breakfast and/or lunch, the need to consider several factors is essential. A policy and legal framework to safeguard children’s right to adequate food in schools is available now but also limited. However, to operate effectively, there is also needed to consider (1) capacity building and providing technical guidance on education strategies to support the acceptability, ownership, compliance with, and impact of SFGs; (2) guidance on school food procurement; (3) guidance to adapt and support a healthy food environment. In this context, research is needed to validate and pilot the factors needed, in order to assure the buy-in at different levels, and to prepare and propose a detailed plan with clear inputs and outputs to reach certain outcomes and the desired objectives. Somehow, we are also asking the private sector to reach public health goals, so much advocacy and negotiation are also needed.

## 5. Conclusions

This food environmental assessment using mixed methods shows that before the official implementation of the SFGs for the preparation, distribution, and sale of food and beverages within all schools of the National Education System, the food environment was characterized by the high availability of UPFs and SSBs, both outside (public schools) and inside schools (private school), as well as commonly included in lunchboxes across the three schools studied. Additionally, UPFs and SSBs frequently contained sugars, artificial colorants such as red 40, front-warning labels for excess sugars and energy, and packaging in single-use materials, with a lack of functional drinking fountains in public primary schools. Although principals, teachers, and vendors were aware of the SFGs, barriers to compliance remain. These include resistance from parents and students to adopt healthier eating habits, the economic dependence of school cooperatives on the sale of UPFs, and insufficient infrastructure in public schools to support the preparation and sale of nutritionally adequate foods. Together, these findings highlight persistent structural and economic challenges within school food environments. Overall, the results underscore the need to strengthen monitoring, training, and active communication between school authorities, families, and vendors, as well as to promote sustainable economic alternatives. Comprehensive strategies are essential to support the effective implementation of policies promoting healthier school nutrition, reducing children’s exposure to unhealthy foods, and advancing toward healthy and sustainable school food environments.

## Figures and Tables

**Table 1:** General characteristics of schools.

School ID	District	Type of School	Date of Enrolled	Extended School Day	Total Children Registered (*n*)	Attend the Day of Evaluation *n* (%)	Number of Food Waste Collected Items *n* (%)	Items Consumed by Student
S1	Álvaro Obregón	Public	14 March 2025	No	469	367 (78.3)	167 (48.4)	0.46
S2	Cuajimalpa de Morelos	Private	25 March 2025	Yes	484	478 (98.8)	118 (34.2)	0.25
S3	Álvaro Obregón	Public	4 April 2025	No	187	175 (93.6)	60 (17.4)	0.34
Total *n* (%)					1140	1020 (89.5)	345 (100)	0.33

**Table 2:** Type of participants interviewed, by school.

Participant	S1	S2	S3	Total
Principal	1	1	1	3
Teachers	3	8	4	15
Food vendors	2	2	1	5
Total	6	11	6	23

## Data Availability

The data presented in this study are available on request from the corresponding author. The data are not publicly available due to privacy and ethical reasons.

## Supplementary Materials

The following supporting information can be downloaded at https://www.mdpi.com/article/10.3390/children13010088/s1, Figure S1: Photographic record of Food offered inside and outside of schools; Figure S2: Photographic record of Access to drinking water; Figure S3: Photographic record of Lunch boxes visual analysis; Figure S4: Photographic record of Food waste audit assessment; Figure S5: Ingredients information of food waste; Figure S6: Types of colorants found in the packages of food waste; Figure S7: Front warning labels in food waste; Figure S8: Packaging materials in food waste.

## Abbreviations

The following abbreviations are used in this manuscript:

ANSA	National Agreement for Nutritional Health
ENSANUT	National Health and Nutrition Survey
FAO	Food and Agriculture Organization of the United Nations
HBM	Health Belief Model
OW/OB	Overweight and obesity
SFGs	School Food Guidelines
SSBs	Sugar-sweetened beverages
TPB	Theory of Planned Behavior
UPFs	Ultra-processed foods
WHO	World Health Organization
