# Supplementary material for: Food Environment Assessment in Primary Schools Before the Implementation of Mexico’s 2025 School Food Guidelines: A Mixed Method Analysis
Source: Children (Basel). 2026 Jan 6;13(1):88. doi: 10.3390/children13010088 (PMC12840457; doi:10.3390/children13010088)

**Supplementary Figure S3.** Photographic record of Lunch boxes visual analysis

**S1**

There was no photographic record at the S1 school.

**S2**

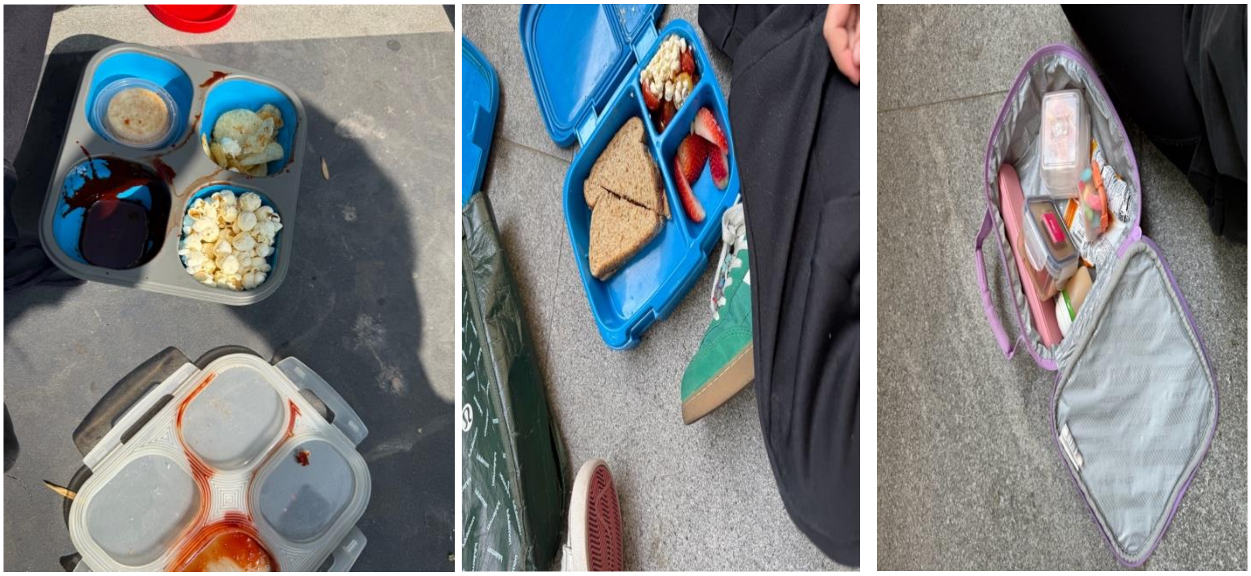

**S3**

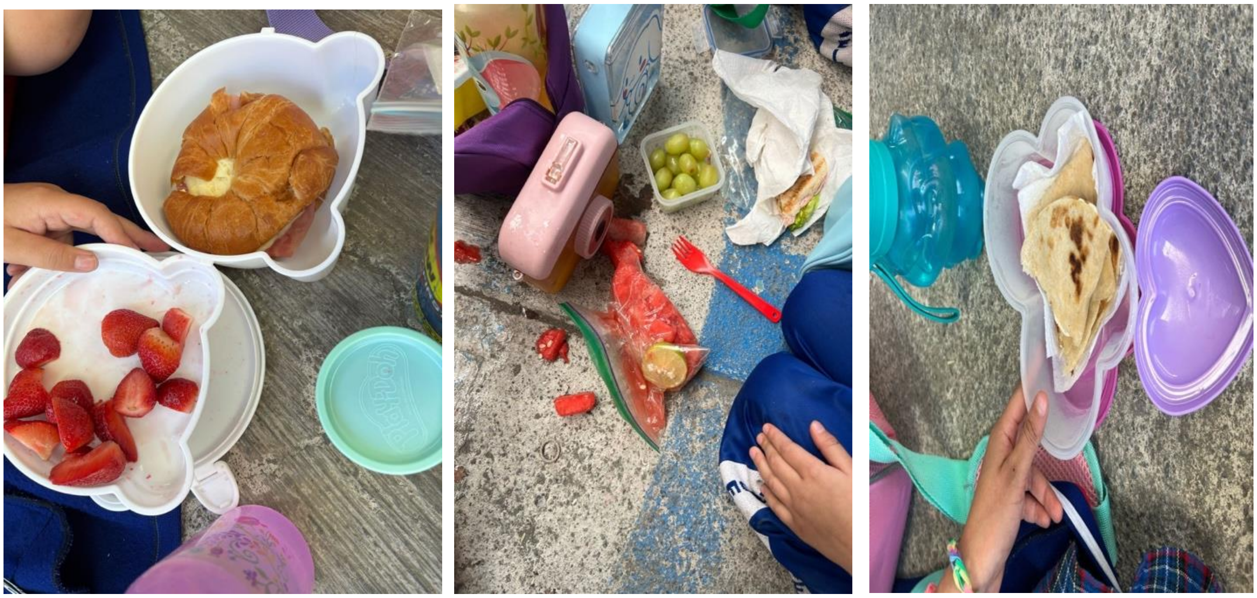

Supplement: Supplementary file 1 [file children-13-00088-s001.zip › S3.pdf]
