# Supplementary material for: Food Environment Assessment in Primary Schools Before the Implementation of Mexico’s 2025 School Food Guidelines: A Mixed Method Analysis
Source: Children (Basel). 2026 Jan 6;13(1):88. doi: 10.3390/children13010088 (PMC12840457; doi:10.3390/children13010088)
Supplement: Supplementary file 1 [file children-13-00088-s001.zip › S5.pdf]

Supplementary Figure S5. Ingredients information of food waste

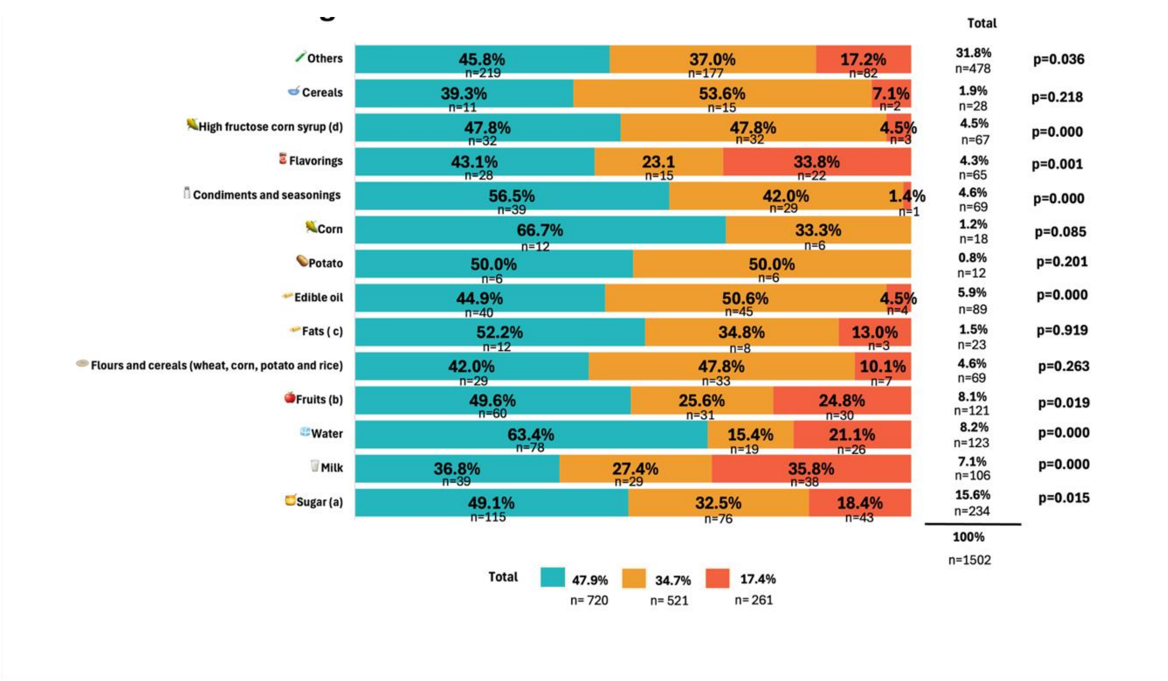

a: including sugars intrinsic to food and added sugars like cane sugar; b: including pulps, juices, purees, fruit preparations, fruit water, whole fruit, and fruit concentrates; including vegetable oil, vegetable fat, and margarines; d: including high fructose corn syrup and corn syrup. Fisher's exact test was applied.
