# Supplementary material for: Food Environment Assessment in Primary Schools Before the Implementation of Mexico’s 2025 School Food Guidelines: A Mixed Method Analysis
Source: Children (Basel). 2026 Jan 6;13(1):88. doi: 10.3390/children13010088 (PMC12840457; doi:10.3390/children13010088)
Supplement: Supplementary file 1 [file children-13-00088-s001.zip › S6.pdf]

Supplementary Figure S6. Types of colorants found in the packages of food waste

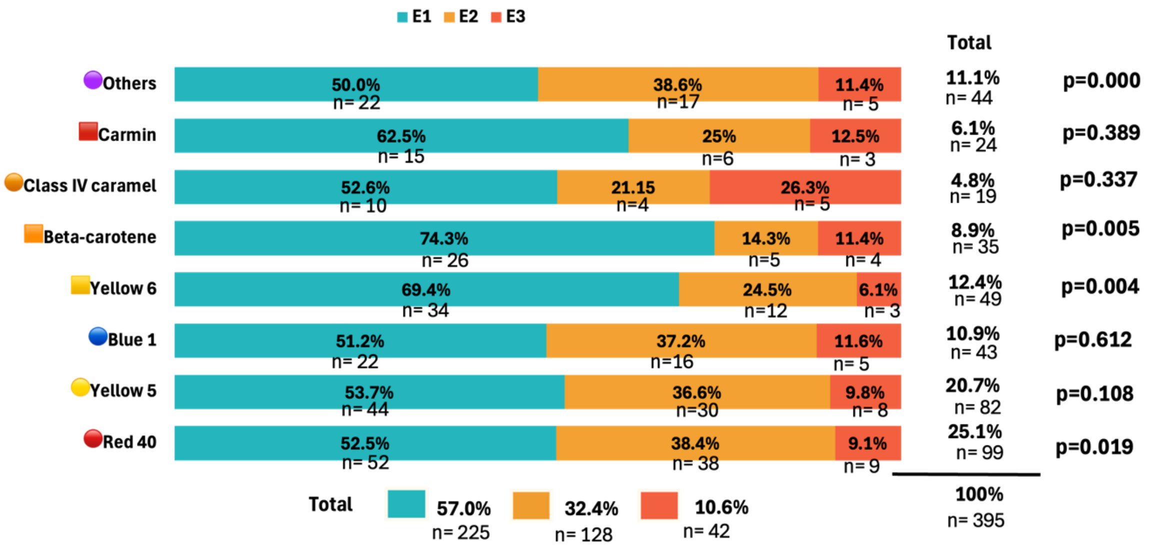

Red 40: including allura AC, allura, INS 129, CI16035; Blue 1: including bright blue FC, INS 33; Yellow 6: including sunset FCF, sunset; Beta-carotene: including carotene; Class IV caramel IV: including INS 150d; Carmin: including Cochineal red E120, carmine E120. Fisher's exact test was applied.
