# Supplementary figures and images for: Food Environment Assessment in Primary Schools Before the Implementation of Mexico’s 2025 School Food Guidelines: A Mixed Method Analysis
Source: Children (Basel). 2026 Jan 6;13(1):88. doi: 10.3390/children13010088 (PMC12840457; doi:10.3390/children13010088)

**Supplementary Figure S1.** Photographic record of Food offered inside and outside of schools

S1

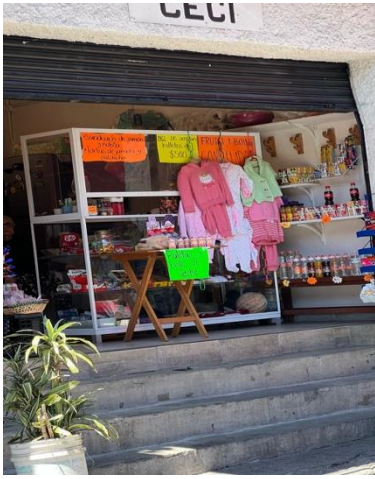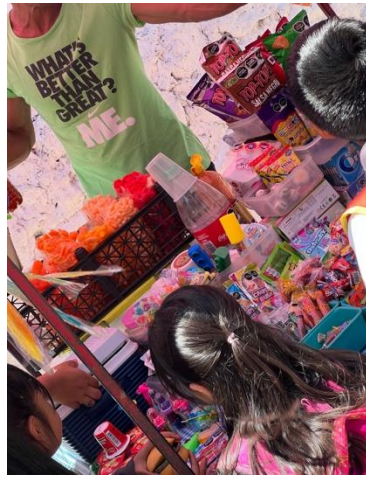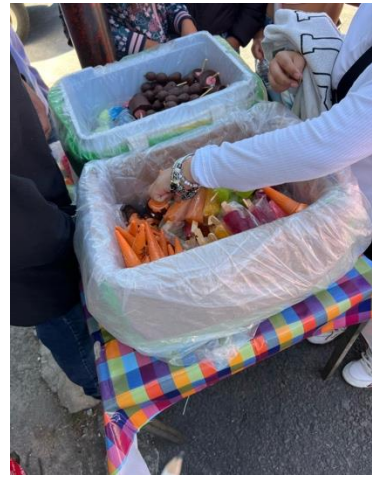

S2

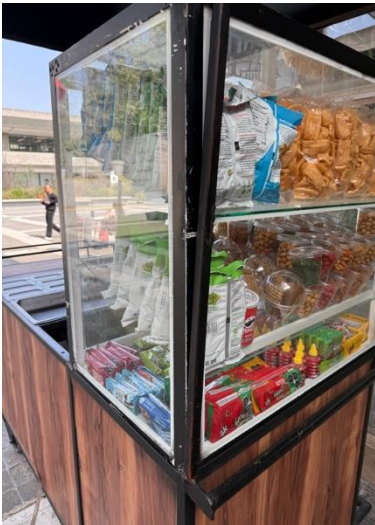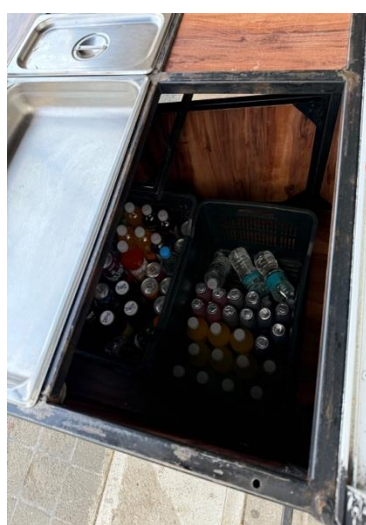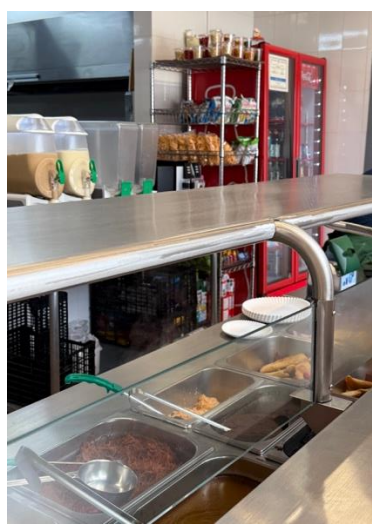

S3

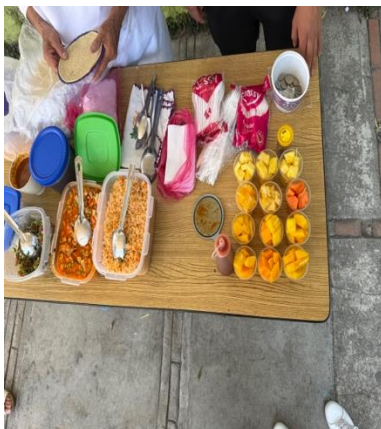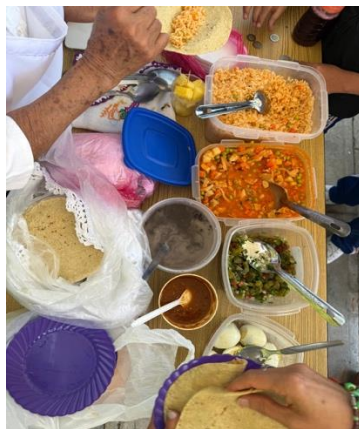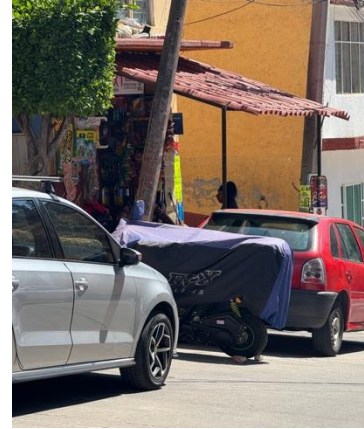

Supplement: Supplementary file 1 [file children-13-00088-s001.zip › S1.pdf]

## Supplementary Figure S2. Photographic record of Access to drinking water

S1

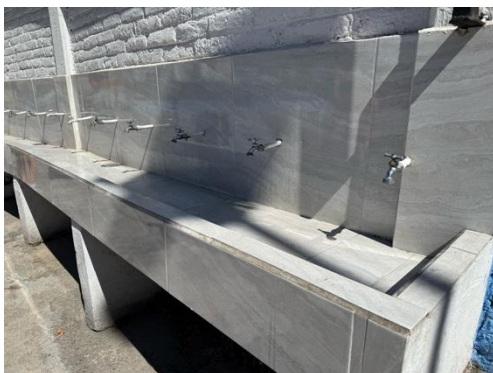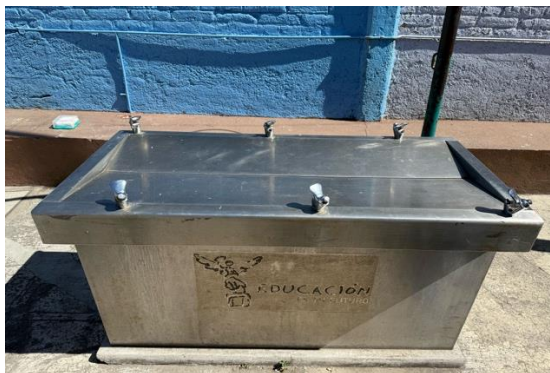

S2

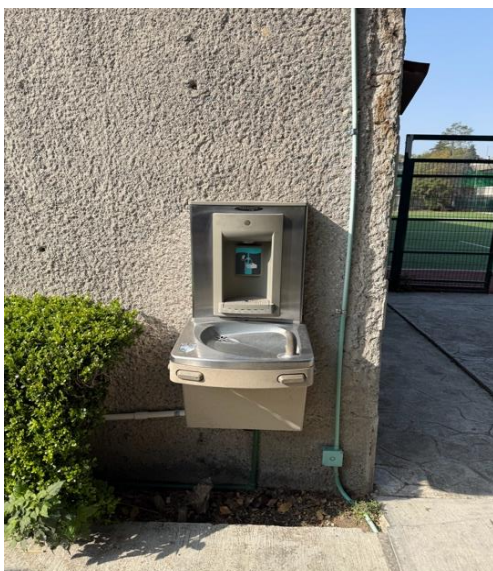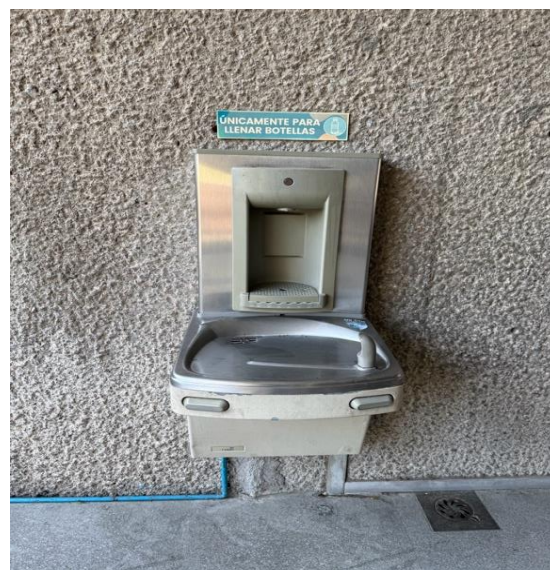

S3

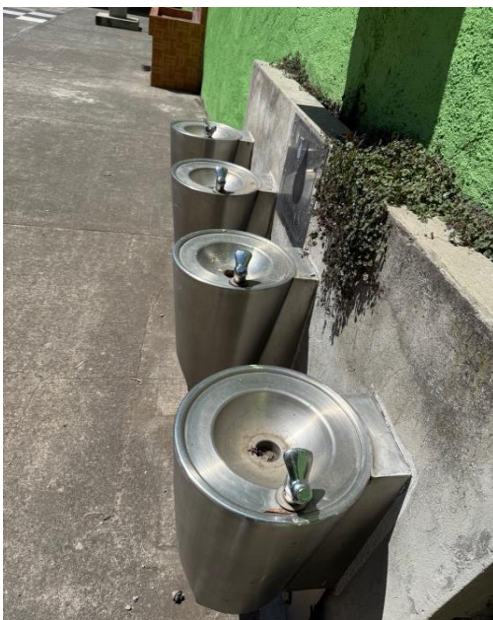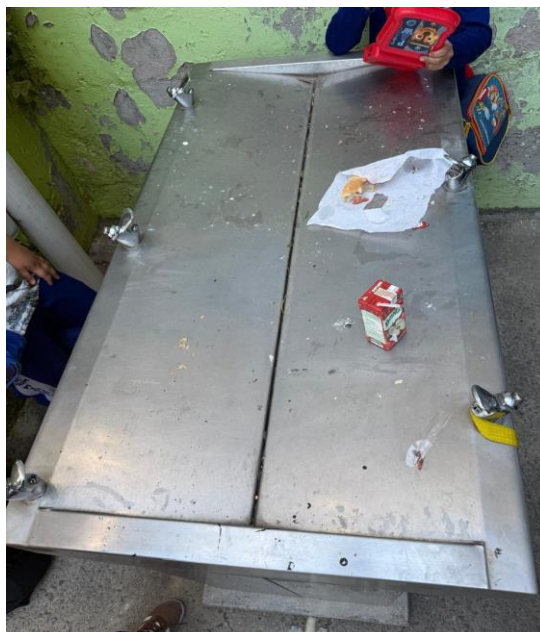

Supplement: Supplementary file 1 [file children-13-00088-s001.zip › S2.pdf]

## Supplementary Figure S4. Photographic record of Food waste audit assessment

S1

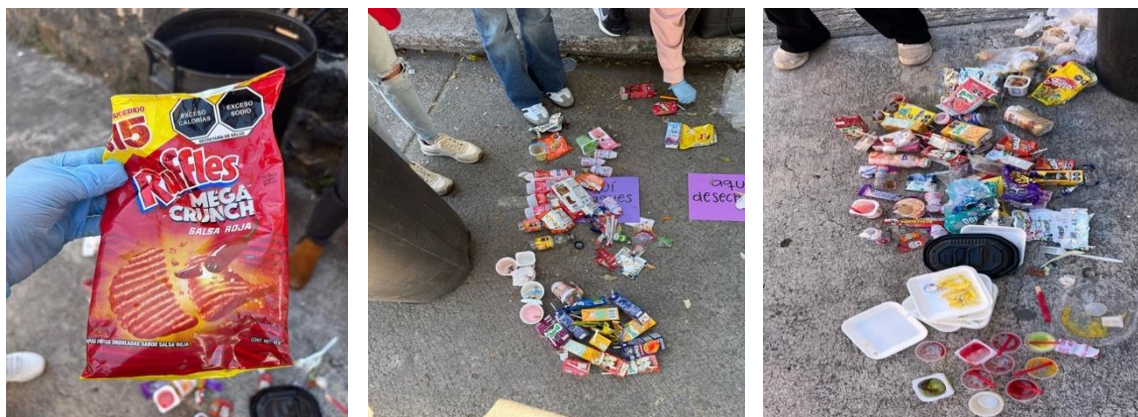

S2

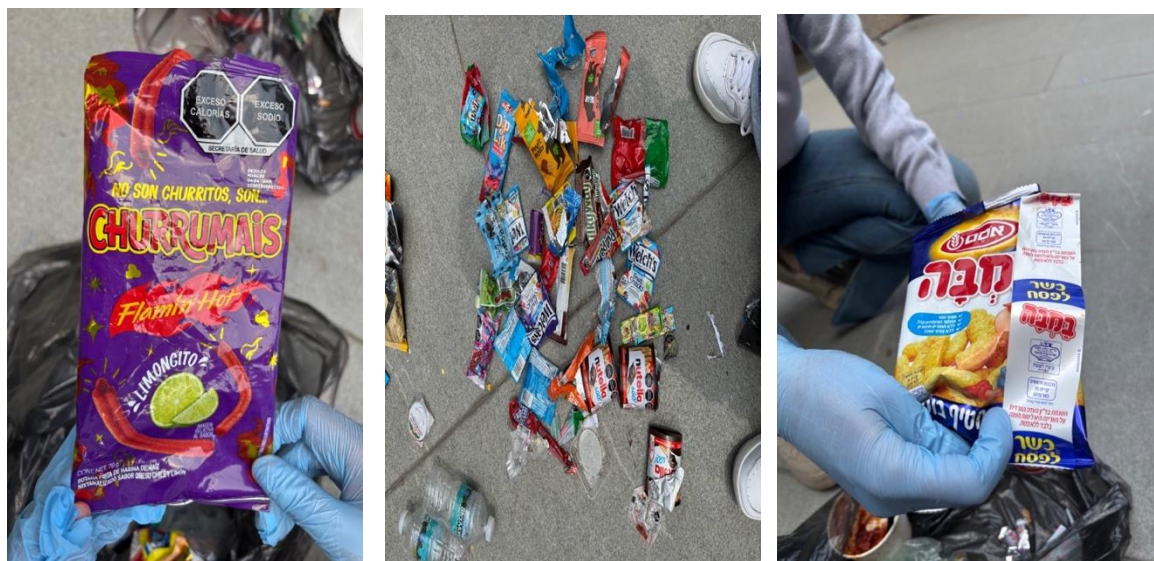

S3

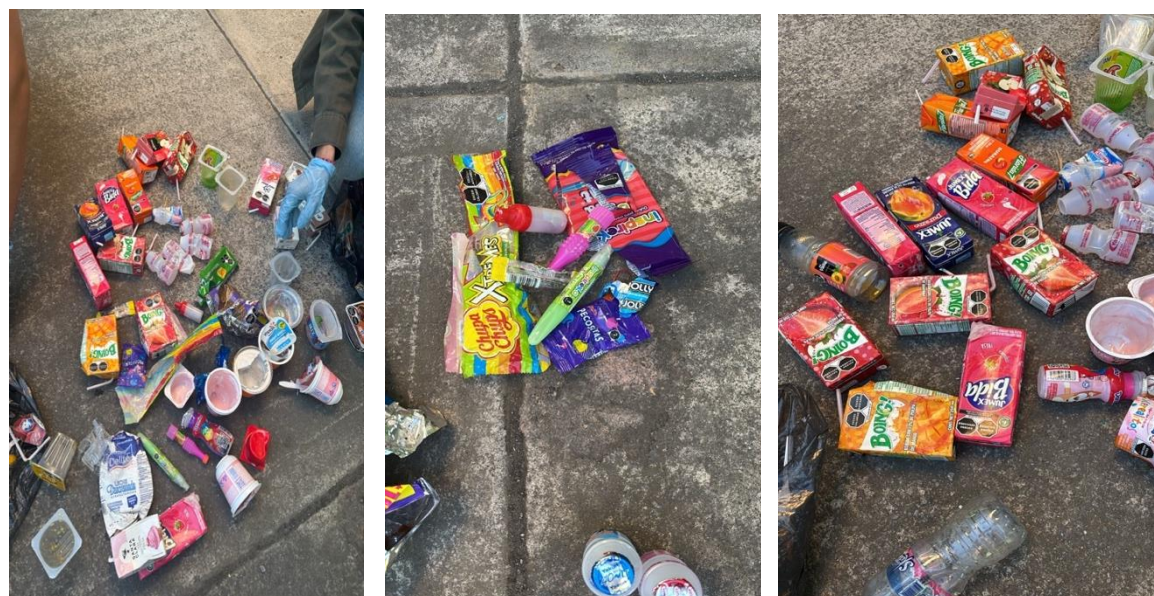

Supplement: Supplementary file 1 [file children-13-00088-s001.zip › S4.pdf]

## Supplementary Figure S7. Front warning labels in food waste

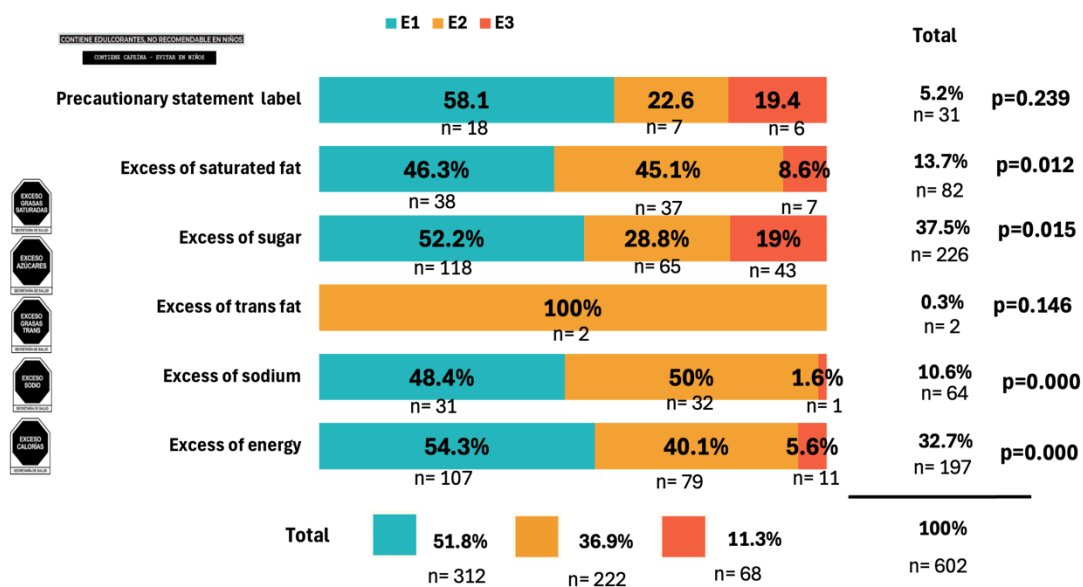

Fisher's exact test was applied.

Supplement: Supplementary file 1 [file children-13-00088-s001.zip › S7.pdf]

Supplementary Figure S8. Packaging materials in food waste.

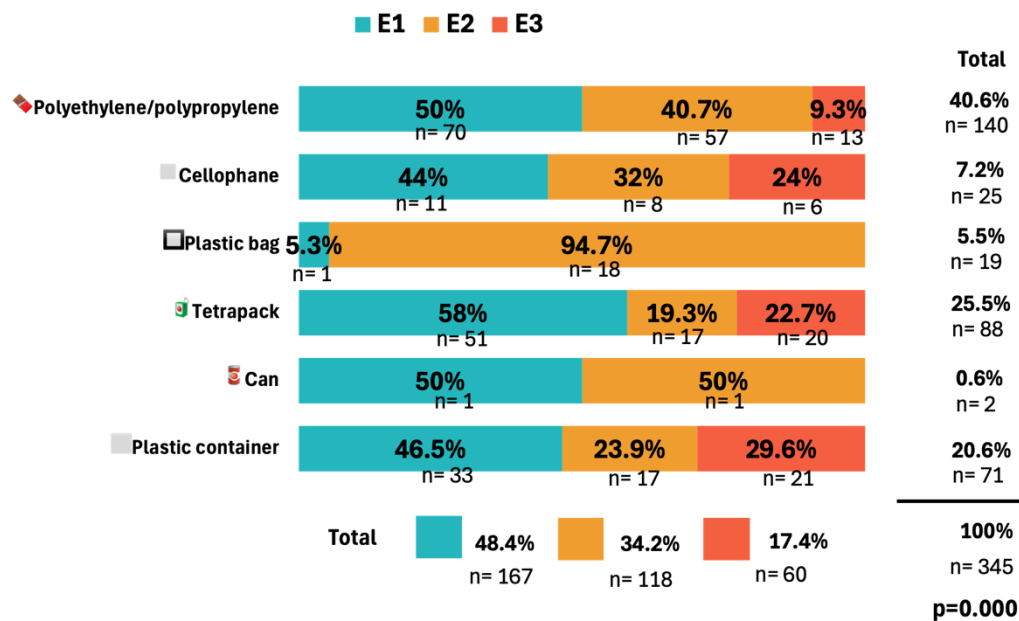

Fisher's exact test was applied.

Supplement: Supplementary file 1 [file children-13-00088-s001.zip › S8.pdf]
